# Effects of transcranial direct current stimulation on the cognitive control of negative stimuli in borderline personality disorder

**DOI:** 10.1038/s41598-018-37315-x

**Published:** 2019-01-23

**Authors:** Lars Schulze, Maren Grove, Sascha Tamm, Babette Renneberg, Stefan Roepke

**Affiliations:** 1 Clinical Psychology and Psychotherapy, Freie Universität Berlin, Berlin, Germany; 2 Department of Psychiatry and Psychotherapy, Ludwig-Maximilians-University, Munich, Germany; 3 Experimental and Cognitive Neuropsychology, Freie Universität Berlin, Berlin, Germany; 4 Department of Psychiatry, Charité - Universitätsmedizin Berlin, Campus Benjamin Franklin, Berlin, Germany

## Abstract

Borderline personality disorder (BPD) is characterized by impairments in the cognitive control of negative information. These impairments in cognitive control are presumably due to blunted activity of the dorsolateral prefrontal cortex (dlPFC) along with enhanced activations of the limbic system. However, the impact of an excitatory stimulation of the dlPFC still needs to be elucidated. In the present study, we therefore assigned 50 patients with BPD and 50 healthy controls to receive either anodal or sham stimulation of the right dlPFC in a double-blind, randomized, between-subjects design. Participants performed a delayed working memory task with a distracter period during which a grey background screen, or neutral, or negative stimuli were presented. This experimental paradigm was first evaluated in a pilot study with 18 patients with BPD and 19 healthy controls. In both studies, patients with BPD showed an impairment of cognitive control when negative distracters were presented in the delay period of a working memory task. However, excitatory stimulation of the right dlPFC did not ameliorate cognitive control of negative stimuli in BPD, which raises questions about the specific role of the right dlPFC for the understanding of BPD psychopathology. Methodological limitations are discussed.

## Introduction

Borderline personality disorder (BPD) is a serious mental disorder characterized by affective disturbances, impulsivity, self-injury, and chronic suicidal tendencies^1,2^. Of particular interest for the understanding of BPD psychopathology are impairments in cognitive control that allow individuals to process and maintain goal-relevant information, while adapting flexibly to changing environmental demands. Cognitive control is particularly important when individuals are presented with salient but irrelevant information that distracts resources from their current tasks, such as the presence of emotionally evocative information. Individual differences in the ability to control such irrelevant information are associated with different aspects of psychosocial functioning and mental health^3–6^.

A multitude of experimental studies investigated cognitive control in BPD. Experimental studies have hitherto mostly illustrated that patients with BPD do not show general deficits in cognitive control e.g.^7–9^. It was rather suggested that pronounced impairments in the cognitive control of *negative* affective material are characteristic for BPD. For instance, patients with BPD show an impaired inhibition of negatively valenced material in comparison to healthy controls^10,11^. Additional findings suggest that patients with BPD are more susceptible to interference from negative, schema-related stimuli^12,13^. The presentation of such negative distracting information was found to enhance response latencies or decrease accuracy scores in patients with BPD compared to healthy controls^9,14,15^. However, findings of an enhanced interference of negative stimuli with cognitive processes in BPD are not unequivocal. Several experimental studies found no valence-specific effects of emotional distracters on cognitive control^10,16,17^.

Functional imaging studies elucidated the neural basis of impaired cognitive control of negative material in BPD. These studies illustrated congruently prefrontal dysfunctions in orbitofrontal and dorsolateral regions of patients with BPD compared to healthy controls^16–22^. A recent meta-analysis concluded that BPD patients’ impairments in the cognitive control of negative stimuli are presumably the result of blunted activity of the dorsolateral prefrontal cortex (dlPFC) along with enhanced activation of the limbic system^23^.

However, despite the centrality of dlPFC abnormalities for neurobiological models of BPD^24^, no study to date has investigated the behavioral effects of an excitatory stimulation of this brain region in BPD. Transcranial direct current stimulation (tDCS) represents a simple and presumably effective way to alter cortical brain activity^25–27^. Beneficial effects of excitatory dlPFC stimulation on executive functioning have been reported for healthy and clinical samples^28^. Notably, experimental studies have also provided promising results that excitatory stimulation of the dlPFC ameliorates cognitive control of aversive stimuli not only in healthy controls^29^, but also in patients with major depression^30^.

In the present study, we investigated whether excitatory stimulation of the right dlPFC (compared to a sham condition) results in an amelioration of cognitive control of negative stimuli in BPD. To this end, participants performed a delayed working memory task with a distracter period during which either a grey background screen, or neutral, or negative stimuli were presented. This task was first evaluated in a pilot study (Study 1). We hypothesized that negative distracters result in prolonged response latencies in patients with BPD compared to healthy controls. The main study (Study 2) assessed the effects of anodal stimulation of the right dlPFC in patients with BPD and healthy controls. We expected an amelioration of cognitive control of negative stimuli in BPD during excitatory stimulation of the right dlPFC compared to a sham condition.

## Study 1

### Research questions

This pilot study investigated whether the presentation of negative distracters in a delayed working memory task interferes with behavioral performance in patients with BPD^9,14^. More specifically, we expected prolonged response latencies in patients with BPD compared to healthy controls. Furthermore, we explored whether valence-dependent interference with behavioral performance in patients with BPD is modulated by the length of the distracter presentation (i.e. interference duration).

### Methods

#### Participants

We enrolled a convenience sample of 20 patients with borderline personality disorder and 20 healthy controls in this study. One patient did not finish the experimental paradigm. Another patient and one healthy control had a general hit rate below 65% indicating insufficient engagement in the experimental task; both participants were excluded from the analysis. Thus, the final sample comprised 18 patients with BPD and 19 healthy controls.

Healthy controls were recruited via public advertising. BPD patients were recruited at the Department of Psychiatry, Charité - Universitätsmedizin Berlin. At the time of study participation, patients were part of a specialized psychotherapeutic inpatient treatment program for BPD. These patients were on a waiting list prior to treatment and none of them was admitted for acute psychiatric care. All participants underwent diagnostic interviews with German versions of the Mini-International Neuropsychiatric Interview for DSM-IV Axis-I Mental Disorders^31^ and the Structured Clinical Interview for DSM-IV Axis-II Personality Disorders^32^. Clinical psychologists holding a master’s degree in psychology conducted the clinical interviews. Interviewers were trained in the use of these instruments and supervised by the senior author. We did assess interrater reliabilities of this procedure for SCID-II personality disorder diagnoses in our research group^33^. We found acceptable interrater reliabilities of κ = 0.82 for a diagnosis of BPD, and acceptable internal consistencies with Cronbach’s α = 0.88 for the sum of BPD criteria. Participants recruited via media advertisements were initially screened by telephone, before undergoing the clinical interview in the lab directly before the experiment. Furthermore, participants were screened regarding basic cognitive abilities (LPS-4)^34^. Healthy controls were only included if they did not take any psychotropic medication and had neither a current nor a lifetime diagnosis of any mental or neurological disorders (e.g., traumatic diseases of the central nervous system). Exclusion criteria for BPD patients were comorbid diagnosis of past or present psychotic disorder, bipolar disorder, cognitive disorders (e.g., delirium, dementia), or neurological disorders as well as substance-associated disorders within three months prior to study participation.

Patients with BPD and healthy controls did not differ with regard to basic socio-demographic variables, such as age (BPD: M = 29.67, SD = 8.85; HC: M = 33.05, SD = 7.15; W = 118.5, p = 0.11), gender (BPD: 15 female, 3 male; HC: 18 female, 1 male; p = 0.34, Fisher’s exact test), and intellectual abilities (raw score LPS-4, BPD: M = 26.11, SD = 5.04; HC: M = 26.32, SD = 3.30; t(29.08) = 0.15, p = 0.89). A total of 13 patients received psychotropic medication at the time of the study. The most frequent (n > 2) comorbid mental disorders in our sample were major depression (n = 6), posttraumatic stress disorder (n = 4), panic disorder (n = 4) as well as avoidant personality disorder (n = 4), and antisocial personality disorder (n = 3).

All participants gave written informed consent prior to participation. The ethics committee of the Charité-Universitätsmedizin Berlin approved the study protocol. The experiment was performed in accordance with the Declaration of Helsinki. The study took place at the Department of Psychiatry, Charité-Universitätsmedizin Berlin between February 2013 and July 2013.

#### Experimental Paradigm

Participants performed a delayed working memory task^14,35^. Each trial started with a fixation cross (1000 ms), followed by the presentation of six target letters (1500 ms), which participants were asked to memorize. After a variable distracter period (i.e., interference duration of 1000, 2000, or 4000 ms), participants were presented a recognition display (until a response was made) and had to decide whether the presented letter was part of the initial set of letters. In half of the trials, the recognition display contained a previously presented target. Participants were asked to respond as quickly and accurately as possible.

The distracter period of the experimental paradigm was manipulated with regard to the factors valence (grey background screen, or neutral, or negative stimuli) and interference duration (1000, 2000, or 4000 ms). Neutral and negative affective stimuli were selected from the International Affective Picture System^36^. Valence ratings (rated from 1 - very negative to 9 - very positive) were M = 5.01, SD = 0.35 for neutral stimuli, and M = 2.33, SD = 0.42 for negative stimuli. Arousal ratings (rated from 1 - not arousing at all to 9 - highly arousing) were M = 3.75, SD = 0.86 for neutral stimuli, and M = 5.86, SD = 0.82 for negative stimuli. Neutral and negative stimuli were matched with regard to luminance and visual complexity as determined from jpeg size in bytes (all p’s > 0.40)^37^.

The experiment contained 180 trials, divided into nine blocks with 20 trials each. Each block contained a unique experimental condition (e.g., negative stimuli presented for 1000 ms). Visual stimuli in these blocks were matched regarding valence, arousal, luminance, and visual complexity (all p’s > 0.55). Experimental blocks were presented in pseudo-random order.

The experiment was conducted on a standard notebook connected with a 15-inch screen (screen resolution of 1024 × 768). We also recorded participants’ eye movements and skin conductance, but data will not be reported here. Presentation of visual stimuli and collection of behavioral data was realized using PsychoPy^38^. All participants underwent a training session to familiarize them with the experimental task.

#### Statistical Analyses

Reaction times (RT) from erroneous responses and below <300 ms were filtered. Next, response latencies below or above three times the interquartile range from each individual’s median value in each experimental condition were excluded from the analysis (BPD: 15.2%, HC: 13.5%). Based on the remaining trials, median response times were calculated for each condition.

It was also analyzed whether the percentage of accurate responses and response latencies for accurate responses were correlated. There was no significant association in the control group (r = 0.05, p = 0.75), but we found a negative correlation of r = −0.38, p = 0.03 in the BPD group. Less accurate responding was associated with longer response latencies in BPD participants.

Our primary analyses focused on response times as a dependent variable. First, RTs were subjected to a mixed-design analysis with the within-subject factors valence and interference duration, and the between-subject factors group. Significant interactions were followed by simple effect analyses. Second, response latencies from neutral conditions were subtracted from response latencies for negative conditions. The respective difference score was entered into a Welch t-test with the factor group and subsequent one-sample t-tests per group. Finally, condition-wise hit rates were analyzed.

All analyses were conducted with the *System for Statistical Computation and Graphics R*^39^, applying the packages *afex*^40^, and *emmeans*^41^. Statistical tests were conducted at a 5% significance level. Note, we applied Greenhouse-Geisser corrections regardless of violations of sphericity.

#### Data availability

The full data set, syntax, and statistical results are available at https://osf.io/g43bh/iew_only=f647fa67773041669f0a670c234dd150.

### Results

#### Response Times

As hypothesized, our analysis highlighted a significant interaction of valence and diagnostic group (F(1.90, 66.57) = 3.84, p = 0.03, generalized η^2^ = 0.01). Follow-up tests of estimated marginal means showed enhanced response latencies in patients with BPD compared to the control group only when negative stimuli were presented (t(48.93) = 2.42, p = 0.02, r = 0.33; BPD: M = 1383 ms, SE = 52; HC: M = 1206 ms, SE = 51). No group differences were observed for neutral stimuli or the control condition (all p’s > 0.21).

Except for a significant main effect of valence (F(1.90, 66.57) = 13.05, p < 0.001, generalized η^2^ = 0.04), there were no further main effects or interactions in this analysis (all p’s > 0.14). Descriptive results are presented in Supplementary Table 1.

**Figure 1:**
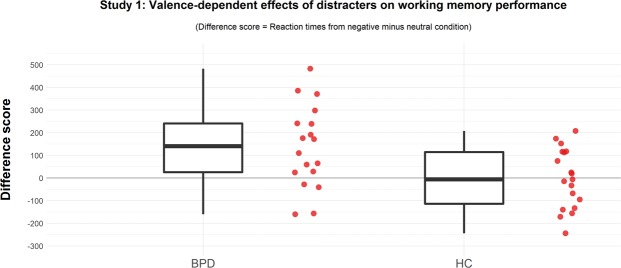
Separate boxplots and individual results of response latencies for trials with negative distracters controlled for response latencies to neutral stimuli for patients with BPD and healthy controls.

Our second (complementary) analysis illustrated that patients with BPD show a stronger interference of negative stimuli (in comparison to healthy controls) even when controlling for response latencies to neutral stimuli (t(30.83) = 2.68, p = 0.008, r = 0.43). This valence-dependent increase in response latencies differed significantly from zero in patients with BPD (M: 137 ms [SD: 181], t(17) = 3.20, p < 0.001), but not in healthy controls (M: −3 ms [SD: 131], t(18) = −0.10, p = 0.92). See Fig. 1 for a visualization.

#### Hit Rates

Analyses of hit rates showed that accuracy scores were significantly lower in the BPD group compared to healthy controls (F(1,35) = 4.60, p = 0.04, generalized η^2^ = 0.06; BPD: M = 78.56%, SE = 16.93; HC: M = 83.70%, SE = 16.93). Furthermore, there was a main effect of interference duration (F(1.52,53.25) = 8.03, p = 0.002, generalized η^2^ = 0.03). Accuracy scores were significantly higher for 1000 ms (M = 83.41%, SE = 13.44) compared to 2000ms (M = 80.76, SE = 13.44, p = 0.04), and 4000 ms (M = 79.21, SE = 13.44, p < 0.001), whereas accuracy for 2000 ms and 4000 ms did not differ significantly (p = 0.45). Descriptive results are presented in Supplementary Table 2.

### Summary of Study 1

In line with our hypotheses, the main analyses of response latencies showed that patients with BPD were more easily distracted by negative stimuli than healthy controls. We did not find empirical evidence that this valence-dependent interference with working memory processes in BPD was further modulated by the length of the interference duration. With respect to accuracy rates, we observed that BPD patients were less accurate than healthy controls and that hit rates were lower for longer presentation times.

In sum, our results reinforce previous findings that patients with BPD are more susceptible to interference by negative stimuli.

## Study 2

### Research questions

The results of our pilot study demonstrate that the experimental paradigm is able to assess valence-dependent impairments of cognitive control in BPD. In Study 2, we thus investigated whether excitatory stimulation of the right dlPFC (compared to a sham condition) results in an attenuation of this valence-dependent interference effect in patients with BPD, i.e., we examined the three-way interaction of valence by group and stimulation.

### Methods

#### Participants

Fifty in- and outpatients with BPD and 50 healthy controls matched for age, gender, and intelligence were enrolled in this study. Power analyses yielded a total sample size of 80 individuals for the detection of a significant interaction with an assumed effect size of 0.06 (partial η^2^ = 0.06, f(U) = 0.25) and a power of 80%. To account for potential data loss, we aimed for a sample size of 25 individuals per group (in total 100 participants). Two patients had a mean hit rate below 65% and were excluded from all statistical analyses. Thus, the final sample comprised 48 patients with BPD and 50 healthy controls. Demographic and clinical characteristics are presented in Table 1.

**Table 1:** Demographic and clinical characteristics of BPD patients and control participants, separated by sham and verum stimulation of the right dorsolateral prefrontal cortex.

	Borderline personality disorder		Healthy controls		Statistics
	Sham stimulation (n = 25)	Verum stimulation (n = 23)	Sham stimulation (n = 26)	Verum stimulation (n = 24)	
**Demographical characteristics**					
Age	32.56 (8.57)	31.61 (8.50)	30.50 (7.45)	32.29 (8.41)	all p’s > 0.40^a^
LPS-4	28.60 (4.92)	26.26 (5.88)	26.88 (5.15)	27.63 (5.76)	all p’s > 0.15^a^
Gender	2 male, 23 female	2 male, 21 female	3 male, 23 female	2 male, 22 female	all p’s > 0.65^b^
Medication intake	16 yes, 9 no	12 yes, 11 no			p > 0.55^c^
**Clinical characteristics**					
BSL-95	2.12 (0.57)	2.02 (0.77)	0.29 (0.17)	0.41 (0.35)	p < 0.001^a^ (diagnostic group)
BDI	25.38 (9.51)	26.52 (11.79)	2.88 (3.68)	3.63 (4.84)	p < 0.001^a^ (diagnostic group)
GSI	2.03 (0.72)	1.94 (0.75)	0.17 (0.19)	0.25 (0.32)	p < 0.001^a^ (diagnostic group)
ALS	74.52 (25.96)	66.02 (27.87)	139.04 (24.36)	134.53 (24.22)	p < 0.001^a^ (diagnostic group)
DERS	132.36 (20.49)	124.22 (22.48)	61.50 (11.28)	65.69 (16.13)	p < 0.001^a^ (diagnostic group)

ALS – Affective Lability Scale, BDI – Beck Depression Inventory, BSL-95 – Borderline Symptom List, DERS – Difficulties in emotion regulation scale, GSI – Global Severity Index, LPS-4 - Leistungspruefsystem, subtest 4.

^a^Based on an univariate general linear model with the factors: group (BPD and HC), and stimulation (sham and verum); ^b^based on a loglinear analysis with the factors group (BPD and HC), and stimulation (sham and verum) – please note the main effect of gender is significant (p < 0.001); ^c^based on Pearsons Chi-Square-Test with the factor stimulation (sham and verum)

Healthy controls and patients with BPD were recruited via public advertising. BPD patients were also recruited at the Department of Psychiatry, Charité – Universitätsmedizin, Berlin.

All participants underwent diagnostic screening with German versions of the Structured Clinical Interview for DSM-IV Axis-I Mental Disorders and Axis-II Personality Disorders^32,42^. Clinical psychologists holding at least a bachelor’s degree in psychology conducted the clinical interviews. Interviewers were trained in the use of these instruments and supervised by the senior author. We did assess interrater reliabilities of this procedure for SCID-II personality disorder diagnoses in our research group^33^. We found acceptable interrater reliabilities of κ = 0.82 for a diagnosis of BPD, and acceptable internal consistencies with Cronbach’s α = 0.88 for the sum of BPD criteria. Participants recruited via media advertisements were initially screened by telephone, before undergoing the clinical interview in the lab directly before the experiment.

We used the exclusion criteria applied in Study 1, but additionally excluded participants with possible tDCS contraindications, such as a cardiac pacemaker, metal in or around the head, pregnancy, or tattoos or scarred skin on the scalp or left deltoid muscle. Furthermore, BPD patients with a current diagnosis of a major depressive episode were also excluded from study participation. The most frequent current comorbid mental disorders (n > 2) were posttraumatic stress disorder (n = 19), eating disorders including anorexia and bulimia nervosa (n = 14), social anxiety disorder (n = 4), substance abuse (n = 3), and paranoid personality disorder (n = 3).

All participants gave written informed consent prior to participation. The ethics committee of the Charité-Universitätsmedizin Berlin approved the study protocol. The experiment was performed in accordance with relevant guidelines and regulations. The study took place at the Department of Psychiatry, Charité-Universitätsmedizin Berlin between January 2016 and June 2017.

#### Transcranial Direct Current Stimulation

Participants were pseudo-randomly assigned to receive either sham or verum stimulation of the right dorsolateral prefrontal cortex within a double-blind, between-subjects design. Direct electrical current was applied by a saline-soaked pair of surface sponge electrodes with a surface of 35 mm² connected to a battery-driven constant current stimulator (DC-Stimulator, NeuroConn GmbH, Ilmenau, Germany). For anodal stimulation of the right dlPFC, the electrode was positioned over F4 according to the 10–20 international system for EEG electrode placement^43^. The cathode was placed on the left deltoid muscle.

During active stimulation a constant current of 1.0 mA was applied for the duration of the experimental paradigm (or a maximum of 20 minutes). To mimic the sensation of tDCS in the sham condition, the current was ramped up and down for 30 seconds respectively at the beginning and end of the experimental session. In the sham condition the stimulator was turned off during the experiment. The stimulation device contained a study mode for double-blind trials. The principal investigator generated numeric codes for active and sham stimulation sessions prior to the experimental sessions. Sequences were generated with in-house functions based on randperm (Matlab). The experimenter entered these preassigned codes and was unaware of the experimental condition.

Participants were asked for the presence of possible side effects of the stimulation. Statistical analyses showed that perception of tingling or burning sensations, pain under the electrodes, light flashes during the stimulation, or headaches and nausea after the stimulation did not differ between sham and verum stimulation of the right dlPFC (all p’s > 0.1). A subsample (n = 44) was asked to guess which stimulation condition they were assigned to. Participants mainly assumed to have received verum stimulation of the right dlPFC (sham condition: 70.83%, verum condition: 75.00%), but groups did not differ significantly.

#### Experimental Paradigm

We used the same experimental procedures as described in Study 1.

#### Statistical Analyses

We used the same statistical procedures as described in Study 1. In short, median response times were calculated after outlier correction (BPD sham: 14.7%, BPD verum: 13.9%, HC sham: 13.4%, HC verum: 13.4%). There were no significant correlations between the percentage of accurate responses and response latencies for accurate responses in the experimental groups (r’s: −0.04 to 0.01, p’s: 0.78–1).

RTs were subjected to a mixed-design analysis with the within-subject factors valence and interference duration, and the between-subject factors group and stimulation. Furthermore, difference scores (negative - neutral condition) were entered into a univariate analysis with the factors stimulation and diagnostic group. Finally, accuracy rates were analyzed with a mixed-design analysis comprising the within-subject factors valence and interference duration, and the between-subject factors group and stimulation.

#### Data Availability

 Hypotheses, sample size, exclusion criteria (i.e., based on general accuracy scores), and statistical analyses were pre-registered. The pre-registration, full data set, syntax, and statistical results are available at https://osf.io/g43bh/?view_only=f647fa67773041669f0a670c234dd150. At the request of this journal, Study 2 was registered as a clinical trial after the initial submission of the manuscript (16/08/2018; clinicaltrials.gov: NCT03636139). This also applies to the research protocol and the CONSORT checklist provided in the supplementary materials.

### Results

#### Main analyses

Response Times: As predicted, the analysis showed a significant interaction of valence and diagnostic group (F(1.93, 180.97) = 5.38, p = 0.006, generalized η^2^ = 0.006). Follow-up tests of estimated marginal means showed that BPD patients were significantly slower than healthy controls when presented with negatively valenced stimuli (t(116.75) = 2.54, p = 0.012, r = 0.23). No group differences were found for neutral stimuli or the baseline condition (all p’s > 0.26).

In contrast to our hypothesis, this valence by diagnostic group effect was not further modulated by an excitatory stimulation of the right dlPFC (F(1.93, 180.97) = 1.18, p = 0.31, generalized η^2^ = 0.001). Rather we found a significant, but unpredicted, three-way interaction of interference duration, diagnostic group, and stimulation (F(1.97, 185.35) = 4.24, p = 0.02, generalized η^2^ = 0.003). Follow-up tests showed neither significant differences between diagnostic groups at specific interference durations (all p’s > 0.09), nor between stimulation conditions (all p’s > 0.14). Furthermore, main effects of valence (F(1.93, 180.97) = 31.49, p < 0.001, generalized η^2^ = 0.03), and interference duration (F(1.97, 185.35) = 14.83, p < 0.001, generalized η^2^ = 0.009) were significant. Descriptive results are presented in Supplementary Table 3.

**Figure 2:**
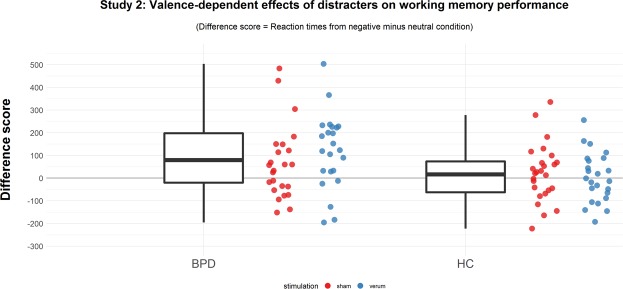
Separate boxplots and individual results of response latencies for trials with negative distracters controlled for response latencies to neutral stimuli for patients with BPD and healthy controls. Individual results are presented separately for the stimulation condition. Please note, one participant had a value of 831 ms and is not presented in the figure. Findings remain significant when excluding this individual from data analysis.

The secondary analysis of individual difference scores showed again a stronger interference of negative stimuli in patients with BPD (in comparison to healthy controls) even when controlling for response latencies to neutral stimuli (F(1,94) = 8.60, p = 0.004, generalized η^2^ = 0.08, see Fig. 2). This additional analysis yielded neither support for a general effect of stimulation nor an interaction of stimulation by diagnostic group (all p’s > 0.47). The valence-dependent increase in response latencies differed significantly from zero in patients with BPD (M: 105 ms [SD: 190], t(47) = 3.85, p < 0.001), but not in healthy controls (12.5 ms [SD: 118], t(49) = 0.75, p = 0.46).

Hit Rates: Compared to healthy controls, percentage hit rates of the BPD sample was significantly lower in the delayed working memory task (BPD: 82.28 (10.03), HC: 85.57 (10.07); F(1,94) = 5.06, p = 0.03, generalized η^2^ = 0.03). Results also showed a main effect of valence (F(1.96, 183.88) = 9.75, p < 0.001, generalized η^2^ = 0.02). Accuracy scores were significantly higher for the control condition (85.63 [10.20]) compared to neutral (83.66 [10.27], p = 0.02), and negative (82.59 [10.27], p < 0.001) stimuli. Accuracy did not differ between neutral and negative stimuli (p > 0.37).

There were no further significant main effects or interactions of the experimental factors (all p’s > 0.07). Descriptive results are presented in Supplementary Table 4.

#### Additional exploratory analyses

BPD groups only: We repeated the statistical analyses presented above, but focused exclusively on patients with BPD. Thus, we did not include the samples of healthy controls in the analyses presented below.

Analyses with response latencies as dependent variable yielded a significant main effect of valence (F(1.73, 79.42) = 22.09, p < 0.001, generalized η^2^ = 0.04) and interference duration (F(1.82, 83.61) = 6.64, p = 0.003, generalized η^2^ = 0.008) as well as a significant interaction of tDCS stimulation and interference duration (F(3.53, 162.35) = 5.42, p = 0.008, generalized η^2^ = 0.007). Follow-up tests showed no significant differences between stimulation conditions at specific interference durations (all p’s > 0.15). The secondary analysis of individual difference scores (negative – neutral) did not show a significant difference between sham and verum conditions (t(44.79) = −0.48, p = 0.64).

Analyses with accuracy rates as dependent variable yielded only a significant main effect of valence (F(1.92, 88.43) = 3.47, p < 04, generalized η^2^ = 0.01).

### Summary

The results show again that negative distracters impair cognitive control in BPD. However, in contrast to our hypotheses, excitatory stimulation of the right dlPFC did not significantly attenuate valence-dependent impairments of cognitive control in patients with BPD.

## Discussion

In this project, we investigated the effects of negative valence as well as of an excitatory stimulation of the right dlPFC on cognitive control in BPD. As predicted, patients with BPD showed an impairment of cognitive control when negative distracters were presented in the delay period of a working memory task. However, in contrast to our hypotheses, excitatory stimulation of the right dlPFC did not ameliorate cognitive control of negative stimuli in BPD.

### Cognitive control in BPD

Response latencies of the BPD group differed significantly from healthy controls only when negative distracters were presented, whereas no group differences were observed during the control condition (i.e. grey background screen) or the presentation of neutral distracters. It is noteworthy that we observed this pattern in two different study samples. Our results reinforce claims that patients with BPD do not exhibit general deficits in cognitive control, but are best characterized by circumscribed impairments in the cognitive control of negatively valenced material.

Notably, enhanced response latencies during the presentation of negative compared to neutral distracters were exclusively found in patients with BPD (contrast negative - neutral, Study 1: M = 137 ms; Study 2: M = 105 ms). Healthy controls did not show valence-dependent behavioral effects (contrast negative - neutral, Study 1: M = −3 ms; Study 2: M = 12.5 ms). This is in line with a recent meta-analysis of the effects of affective information on working memory performance^44^. In that study, only negligible effects of affective task-irrelevant distracters on working memory performance in healthy individuals were found. In contrast, affective stimuli had substantially larger effects in individuals with mental health problems^44^. Negative affective distracters seem to bind cognitive resources in psychopathology, and consequentially impact task-relevant processes.

It remains unclear whether such valence-dependent impairments of cognitive control are uniform or diverse across different forms of psychopathology. This is due to the fact that most experimental studies do not compare subjects with different mental disorders. Rather, and admittedly like our work presented here, most studies compared healthy controls and patients with a specific form of psychopathology (e.g., ADHD, BPD, depression). We decided against inclusion of a clinical control group, since our studies focused exclusively on the replication of impaired cognitive control of negative material in BPD, and the modulation of this effect by means of transcranial direct current stimulation. Future studies are needed to provide empirical answers to questions of disorder-specificity or which specific forms of psychopathology, like repetitive negative thinking, or impairments in daily emotion regulation, are associated with valence-dependent impairments of cognitive control^45^. Such studies might also help to disentangle the role of more general factors, such as mental distress. With regard to disorder-specificity, previous studies compared different facets of behavioral impulsivity in BPD and patients with ADHD^46,47^. Recent reviews of these studies concluded that impulsivity in ADHD reflects deficits in general behavioral inhibition, whereas impulsivity in BPD is mainly driven by affective and interpersonal aspects for reviews see^22,48^.

### Effects of transcranial direct current stimulation

Excitatory stimulation of the right dlPFC did neither ameliorate cognitive control of negative stimuli in patients with BPD, nor in the control group. The lack of a stimulation effect in the control group was expected, since these participants usually do not show a valence-dependent modulation of cognitive control in experimental paradigms. However, previous findings led us to assume a modulatory effect of excitatory stimulation of the right dlPFC on the cognitive control of negative stimuli in BPD. This assumption was based on a number of functional neuroimaging studies, which highlighted blunted activity of the dorsolateral prefrontal cortex during negative emotion processing in BPD^23^. A recent update of that earlier meta-analysis yielded again an attenuated functioning of the right dlPFC in patients with BPD^49^. However, this specific prefrontal abnormality might not be consistently replicable in experimental studies as suggested by the additional results of a robustness analysis (i.e. Jackknife analysis). Future studies should are needed to assess which experimental paradigms or patient characteristics contribute to an attenuated activation of the dlPFC in patients with BPD.

There are some limitations of our study that should be considered in the interpretation of this null-finding. First, we used a between-subjects design. In other words, participants were randomly allocated to receive either sham or verum stimulation of the right dlPFC. The decision for a between-subjects design came at the cost of lower statistical power (compared to a within-subjects design), but had the benefit that there are no order or carryover effects between experimental sessions. Such confounds were previously reported in within-subjects studies of tDCS^30^. Our results indicate the absence of large or medium effect sizes with regard to a three-way interaction of valence by group and stimulation. An additional sensitivity analysis (assuming 80% power) yielded an effect size (Cohens D) of ≥0.73 (one-tailed) or ≥0.83 (two-tailed) for a significant group comparison between BPD patients with and without stimulation of the right dlPFC in the present study. Thus, substantially bigger sample sizes or within-subject designs would be needed to establish the absence or presence of smaller effects. Second, the cathodal electrode was positioned on the left deltoid muscle. Extracephalic positioning of the cathodal electrode allowed unambiguous interpretation of anodal tDCS, since results were not confounded by cathodal effects on another brain region. However, extracephalic positioning of the reference electrode affects the stimulation intensity effective at the dlPFC^50^. Future studies might consider to adapt stimulation intensity accordingly. Third, it remains an empirical question to explore whether left-lateralized or bilateral excitatory stimulation of the dlPFC might ameliorate cognitive control in BPD.

After the start of this study, questions were raised about the general effectiveness of tDCS for the manipulation of executive functioning^51,52^. For instance, Medina and Cason (2017) conclude that their analysis shows minimal evidence (at best) that tDCS influences working memory processes. The debate about the effectiveness of tDCS will be further fueled by a recent finding that about 75% of scalp-applied currents are attenuated by soft tissue and skull^53^. The authors of this work state that higher intensity currents than those of conventional protocols would be necessary to affect neuronal circuits. Further work is necessary to provide reliable manipulations of brain activity and to establish a causal role of specific brain abnormalities for the understanding of BPD-related psychopathology.

### Conclusion

In sum, our results illustrate reliable impairments in the cognitive control of negatively valenced material in BPD. However, excitatory stimulation of the right dlPFC by means of tDCS did not ameliorate deficits in cognitive control of negative stimuli in patients with BPD. Further research is needed to understand the specific role of the right dlPFC in BPD.

## Supplementary information

Supplementary Tables

Supplementary Information: CONSORT checklist

Supplementary Information: Research Protocol
