# Supplementary material for: Effects of transcranial direct current stimulation on the cognitive control of negative stimuli in borderline personality disorder
Source: Sci Rep. 2019 Jan 23;9:332. doi: 10.1038/s41598-018-37315-x (PMC6344572; doi:10.1038/s41598-018-37315-x)
Supplement: Supplementary file 1 — Supplementary Tables [file 41598_2018_37315_MOESM1_ESM.docx]

**Supplementary Information of**

**Effects of transcranial direct current stimulation on the cognitive control of negative stimuli in borderline personality disorder**

Lars Schulze*^1^, Maren Grove^2^, Sascha Tamm^3^, Babette Renneberg^1^, Stefan Roepke^4^

1 Clinical Psychology and Psychotherapy, Freie Universität Berlin, Berlin, Germany; 2 Department of Psychiatry and Psychotherapy, Ludwig-Maximilians-University, Munich, Germany; 3 Experimental and Cognitive Neuropsychology, Freie Universität Berlin, Berlin, Germany 4 Department of Psychiatry, Charité - Universitätsmedizin Berlin, Campus Benjamin Franklin, Berlin, Germany

*Supplementary Table 1*: Descriptive results of response latencies in ms for Study 1

|  |  | **BPD** | |  | **HC** | |
| --- | --- | --- | --- | --- | --- | --- |
|  |  | **Mean** | **SD** |  | **Mean** | **SD** |
| Baseline | 1000 | 1204 | 251 |  | 1094 | 210 |
|  | 2000 | 1204 | 250 |  | 1112 | 282 |
|  | 4000 | 1227 | 191 |  | 1155 | 252 |
| Neutral | 1000 | 1313 | 331 |  | 1221 | 245 |
|  | 2000 | 1195 | 187 |  | 1213 | 268 |
|  | 4000 | 1234 | 266 |  | 1198 | 273 |
| Negative | 1000 | 1401 | 361 |  | 1210 | 300 |
|  | 2000 | 1291 | 307 |  | 1221 | 210 |
|  | 4000 | 1459 | 390 |  | 1192 | 207 |
|  |  |  |  |  |  |  |
| **Valence** | *Baseline* | 1212 | 229 |  | 1120 | 246 |
|  | *Neutral* | 1247 | 267 |  | 1211 | 258 |
|  | *Negative* | 1384 | 355 |  | 1208 | 239 |
| **Duration** | *1000* | 1306 | 322 |  | 1175 | 256 |
|  | *2000* | 1230 | 252 |  | 1182 | 256 |
|  | *4000* | 1307 | 308 |  | 1181 | 242 |
| **Group** |  | 1281 | 296 |  | 1179 | 250 |
|  |  |  |  |  |  |  |
| **Difference** |  | 137 | 181 |  | -3 | 131 |
|  |  |  |  |  |  |  |

*Supplementary Table 2*: Descriptive results of hit rates in % for Study 1

|  |  | **BPD** | |  | **HC** | |
| --- | --- | --- | --- | --- | --- | --- |
|  |  | **Mean** | **SD** |  | **Mean** | **SD** |
| Baseline | 1000 | 83.33 | 8.74 |  | 87.11 | 7.13 |
|  | 2000 | 80.56 | 7.25 |  | 83.42 | 10.81 |
|  | 4000 | 77.22 | 11.79 |  | 84.21 | 12.39 |
| Neutral | 1000 | 81.67 | 9.55 |  | 87.11 | 8.22 |
|  | 2000 | 77.22 | 11.66 |  | 83.68 | 9.84 |
|  | 4000 | 75.28 | 10.64 |  | 80.53 | 12.79 |
| Negative | 1000 | 80.56 | 11.23 |  | 80.26 | 11.96 |
|  | 2000 | 75.56 | 13.81 |  | 83.68 | 9.84 |
|  | 4000 | 75.00 | 12.72 |  | 82.63 | 11.83 |
|  |  |  |  |  |  |  |
| **Valence** | *Baseline* | 80.37 | 9.61 |  | 84.91 | 10.29 |
|  | *Neutral* | 78.06 | 10.79 |  | 83.77 | 10.62 |
|  | *Negative* | 77.04 | 12.65 |  | 82.19 | 11.14 |
| **Duration** | *1000* | 81.85 | 9.78 |  | 84.82 | 9.73 |
|  | *2000* | 77.78 | 11.23 |  | 83.60 | 9.99 |
|  | *4000* | 75.83 | 11.56 |  | 82.46 | 12.22 |
| **Group** |  | 78.49 | 11.10 |  | 83.63 | 10.68 |
|  |  |  |  |  |  |  |

*Supplementary Table 3*: Descriptive results of response latencies in ms for Study 2

|  |  | **BPD - Sham** | |  | **BPD - Verum** | |  | **HC - Sham** | |  | **HC – Verum** | |
| --- | --- | --- | --- | --- | --- | --- | --- | --- | --- | --- | --- | --- |
|  |  | **Mean** | **SD** |  | **Mean** | **SD** |  | **Mean** | **SD** |  | **Mean** | **SD** |
| Baseline | 1000 | 1096 | 317 |  | 1099 | 322 |  | 971 | 210 |  | 1012 | 300 |
|  | 2000 | 1103 | 229 |  | 1117 | 292 |  | 1065 | 264 |  | 1043 | 360 |
|  | 4000 | 1070 | 236 |  | 1229 | 327 |  | 1132 | 341 |  | 1129 | 314 |
| Neutral | 1000 | 1100 | 236 |  | 1146 | 280 |  | 1121 | 320 |  | 1168 | 334 |
|  | 2000 | 1187 | 333 |  | 1190 | 291 |  | 1049 | 200 |  | 1121 | 300 |
|  | 4000 | 1205 | 261 |  | 1213 | 275 |  | 1128 | 232 |  | 1188 | 293 |
| Negative | 1000 | 1245 | 401 |  | 1244 | 360 |  | 1099 | 222 |  | 1155 | 351 |
|  | 2000 | 1295 | 417 |  | 1259 | 245 |  | 1096 | 203 |  | 1155 | 340 |
|  | 4000 | 1231 | 403 |  | 1404 | 398 |  | 1168 | 194 |  | 1174 | 278 |
|  |  |  |  |  |  |  |  |  |  |  |  |  |
| **Valence** | *Baseline* | 1090 | 260 |  | 1148 | 315 |  | 1056 | 281 |  | 1061 | 325 |
|  | *Neutral* | 1164 | 279 |  | 1183 | 279 |  | 1099 | 255 |  | 1159 | 306 |
|  | *Negative* | 1257 | 403 |  | 1302 | 343 |  | 1121 | 207 |  | 1161 | 320 |
| **Duration** | *1000* | 1147 | 328 |  | 1163 | 323 |  | 1064 | 261 |  | 1111 | 332 |
|  | *2000* | 1195 | 340 |  | 1189 | 279 |  | 1070 | 222 |  | 1107 | 333 |
|  | *4000* | 1169 | 313 |  | 1282 | 344 |  | 1143 | 260 |  | 1164 | 292 |
| **Group** |  | 1170 | 326 |  | 1211 | 319 |  | 1092 | 250 |  | 1127 | 319 |
|  |  |  |  |  |  |  |  |  |  |  |  |  |
| **Difference** |  | 93 | 213 |  | 119 | 165 |  | 22 | 126 |  | 2 | 109 |
|  |  |  |  |  |  |  |  |  |  |  |  |  |

*Supplementary Table 4*: Descriptive results of hit rates in % for Study 2

|  |  | **BPD - Sham** | |  | **BPD - Verum** | |  | **HC - Sham** | |  | **HC – Verum** | |
| --- | --- | --- | --- | --- | --- | --- | --- | --- | --- | --- | --- | --- |
|  |  | **Mean** | **SD** |  | **Mean** | **SD** |  | **Mean** | **SD** |  | **Mean** | **SD** |
| Baseline | 1000 | 85.80 | 9.21 |  | 86.09 | 9.41 |  | 86.92 | 9.60 |  | 87.08 | 13.43 |
|  | 2000 | 82.60 | 10.12 |  | 83.70 | 8.82 |  | 86.73 | 9.37 |  | 87.92 | 10.31 |
|  | 4000 | 82.00 | 12.83 |  | 82.83 | 9.98 |  | 89.04 | 8.13 |  | 86.46 | 9.50 |
| Neutral | 1000 | 81.40 | 10.85 |  | 85.22 | 9.23 |  | 84.62 | 10.48 |  | 85.21 | 9.26 |
|  | 2000 | 80.20 | 10.15 |  | 83.26 | 9.61 |  | 86.54 | 10.18 |  | 85.00 | 10.53 |
|  | 4000 | 81.40 | 10.85 |  | 80.43 | 11.67 |  | 86.92 | 8.95 |  | 83.33 | 10.49 |
| Negative | 1000 | 81.40 | 10.95 |  | 82.17 | 10.53 |  | 84.81 | 9.11 |  | 85.21 | 9.38 |
|  | 2000 | 79.20 | 8.86 |  | 82.61 | 9.64 |  | 83.65 | 10.15 |  | 82.08 | 11.41 |
|  | 4000 | 80.80 | 11.52 |  | 80.43 | 10.65 |  | 85.96 | 9.59 |  | 82.29 | 11.13 |
|  |  |  |  |  |  |  |  |  |  |  |  |  |
| **Valence** | *Baseline* | 83.47 | 10.81 |  | 84.20 | 9.38 |  | 87.56 | 9.00 |  | 87.15 | 11.06 |
|  | *Neutral* | 81.00 | 10.49 |  | 82.97 | 10.27 |  | 86.03 | 9.82 |  | 84.51 | 10.01 |
|  | *Negative* | 80.47 | 10.40 |  | 81.74 | 10.18 |  | 84.81 | 9.55 |  | 83.19 | 10.63 |
| **Duration** | *1000* | 82.87 | 10.44 |  | 84.49 | 9.74 |  | 85.45 | 9.68 |  | 85.83 | 10.75 |
|  | *2000* | 80.67 | 9.70 |  | 83.19 | 9.23 |  | 85.64 | 9.88 |  | 85.00 | 10.88 |
|  | *4000* | 81.40 | 11.61 |  | 81.23 | 10.69 |  | 87.31 | 8.89 |  | 84.03 | 10.40 |
| **Group** |  | 81.64 | 10.60 |  | 82.97 | 9.95 |  | 86.13 | 9.49 |  | 84.95 | 10.65 |
|  |  |  |  |  |  |  |  |  |  |  |  |  |
