# Supplementary material for: Effects of transcranial direct current stimulation on the cognitive control of negative stimuli in borderline personality disorder
Source: Sci Rep. 2019 Jan 23;9:332. doi: 10.1038/s41598-018-37315-x (PMC6344572; doi:10.1038/s41598-018-37315-x)
Supplement: Supplementary file 3 — Supplementary Information: Research Protocol [file 41598_2018_37315_MOESM3_ESM.docx]

**Project summary**

Borderline personality disorder (BPD) is characterized by impairments in the cognitive control of negative information. These impairments in cognitive control are presumably due to blunted activity of the dorsolateral prefrontal cortex (dlPFC) along with enhanced activations of the limbic system. However, the impact of an excitatory stimulation of the dlPFC still needs to be elucidated. In the present study, we assigned 50 patients with BPD and 50 healthy controls to receive either anodal or sham stimulation of the right dlPFC in a double-blind, randomized, between-subjects design. Participants performed a delayed working memory task with a distracter period during which a grey background screen, or neutral, or negative stimuli were presented. Response latencies were our primary dependent variable. The study took place at the Department of Psychiatry, Charité-Universitätsmedizin Berlin between January 2016 and June 2017.

**General information**

- Protocol title: Effects of transcranial direct current stimulation on the cognitive control of negative stimuli in borderline personality disorder
- Principal Investigator: Lars Schulze, Freie Universität Berlin, Clinical Psychology and Psychotherapy, Habelschwerdter Allee 45, 14195 Berlin, Germany, phone: +49-30-838 56446; fax: +49-30-838 4 56446; lars.schulze@fu-berlin.de
- Funding: Lars Schulze is supported by a grant from the German Research Foundation (DFG-SCHU 2961/2-1). The study was not externally funded**.**
- Laboratory**:** Department of Psychiatry, Charité - Universitätsmedizin Berlin, Campus Benjamin Franklin, Hindenburgdamm 30, 12203 Berlin, Germany

**Rationale & background information**

Borderline personality disorder (BPD) is a serious mental disorder characterized by affective disturbances, impulsivity, self-injury, and chronic suicidal tendencies (Ansell, Sanislow, McGlashan, & Grilo, 2007; Lieb, Zanarini, Schmahl, Linehan, & Bohus, 2004). Of particular interest for the understanding of BPD psychopathology are impairments in cognitive control that allow individuals to process and maintain goal-relevant information, while adapting flexibly to changing environmental demands. Cognitive control is particularly important when individuals are presented with salient but irrelevant information that distracts resources from their current tasks, such as the presence of emotionally evocative information. Individual differences in the ability to control such irrelevant information are associated with different aspects of psychosocial functioning and mental health (De Panfilis, Meehan, Cain, & Clarkin, 2013; Nigg et al., 2006; Ochsner & Gross, 2005; Zetsche, Bürkner, & Schulze, 2018).

A multitude of experimental studies investigated cognitive control in BPD. Experimental studies have hitherto mostly illustrated that patients with BPD do not show general deficits in cognitive control (Barker et al., 2015; Jacob et al., 2013; Prehn et al., 2013). It was rather suggested that pronounced impairments in the cognitive control of *negative* affective material are characteristic for BPD. For instance, patients with BPD show an impaired inhibition of negatively valenced material in comparison to healthy controls (Domes et al., 2006; Korfine & Hooley, 2000). Additional findings suggest that patients with BPD are more susceptible to interference from negative, schema-related stimuli (Arntz, Appels, & Sieswerda, 2000; Sieswerda, Arntz, Mertens, & Vertommen, 2007). The presentation of such negative distracting information was found to enhance response latencies or decrease accuracy scores in patients with BPD compared to healthy controls (Krause-Utz et al., 2012; Krause-Utz, Elzinga, et al., 2014; Prehn et al., 2013). Functional imaging studies elucidated the neural basis of impaired cognitive control of negative material in BPD. These studies illustrated congruently prefrontal dysfunctions in orbitofrontal and dorsolateral regions of patients with BPD compared to healthy controls (Holtmann et al., 2013; Lang et al., 2012; Schulze et al., 2011; Sebastian et al., 2014; Silbersweig et al., 2007; Soloff, Abraham, Ramaseshan, Burgess, & Diwadkar, 2017; Wingenfeld et al., 2009). A recent meta-analysis concluded that BPD patients’ impairments in the cognitive control of negative stimuli are presumably the result of blunted activity of the dorsolateral prefrontal cortex (dlPFC) along with enhanced activation of the limbic system (Schulze, Schmahl, & Niedtfeld, 2016).

However, despite the centrality of dlPFC abnormalities for neurobiological models of BPD (Krause-Utz, Winter, Niedtfeld, & Schmahl, 2014), no study to date has investigated the behavioral effects of an excitatory stimulation of this brain region in BPD. Transcranial direct current stimulation (tDCS) represents a simple and presumably effective way to alter cortical brain activity (Nitsche & Paulus, 2000, 2001; Nitsche et al., 2008). Beneficial effects of excitatory dlPFC stimulation on executive functioning have been reported for healthy and clinical samples (Dedoncker, Brunoni, Baeken, & Vanderhasselt, 2016). Notably, experimental studies have also provided promising results that excitatory stimulation of the dlPFC ameliorates cognitive control of aversive stimuli not only in healthy controls (Feeser, Prehn, Kazzer, Mungee, & Bajbouj, 2014), but also in patients with major depression (Wolkenstein & Plewnia, 2013).

In the present study, we investigated whether excitatory stimulation of the right dlPFC (compared to a sham condition) results in an amelioration of cognitive control of negative stimuli in BPD. To this end, participants performed a delayed working memory task with a distracter period during which either a grey background screen, or neutral, or negative stimuli were presented. We expected an amelioration of cognitive control of negative stimuli in BPD compared to a sham condition.

**References**

Ansell, E. B., Sanislow, C. A., McGlashan, T. H., & Grilo, C. M. (2007). Psychosocial impairment and treatment utilization by patients with borderline personality disorder, other personality disorders, mood and anxiety disorders, and a healthy comparison group. *Comprehensive Psychiatry*, *48*(4), 329–336. https://doi.org/10.1016/j.comppsych.2007.02.001

Arntz, A., Appels, C., & Sieswerda, S. (2000). Hypervigilance in borderline disorder: a test with the emotional Stroop paradigm. *Journal of Personality Disorders*, *14*(4), 366–373.

Barker, V., Romaniuk, L., Cardinal, R. N., Pope, M., Nicol, K., & Hall, J. (2015). Impulsivity in borderline personality disorder. *Psychological Medicine*, *45*(9), 1955–1964. https://doi.org/10.1017/S0033291714003079

De Panfilis, C., Meehan, K. B., Cain, N. M., & Clarkin, J. F. (2013). The relationship between effortful control, current psychopathology and interpersonal difficulties in adulthood. *Comprehensive Psychiatry*, *54*(5), 454–461. https://doi.org/10.1016/j.comppsych.2012.12.015

Dedoncker, J., Brunoni, A. R., Baeken, C., & Vanderhasselt, M.-A. (2016). A Systematic Review and Meta-Analysis of the Effects of Transcranial Direct Current Stimulation (tDCS) Over the Dorsolateral Prefrontal Cortex in Healthy and Neuropsychiatric Samples: Influence of Stimulation Parameters. *Brain Stimulation*, *9*(4), 501–517. https://doi.org/10.1016/j.brs.2016.04.006

Domes, G., Winter, B., Schnell, K., Vohs, K., Fast, K., & Herpertz, S. C. (2006). The influence of emotions on inhibitory functioning in borderline personality disorder. *Psychological Medicine*, *36*(8), 1163–1172. https://doi.org/10.1017/S0033291706007756

Feeser, M., Prehn, K., Kazzer, P., Mungee, A., & Bajbouj, M. (2014). Transcranial direct current stimulation enhances cognitive control during emotion regulation. *Brain Stimulation*, *7*(1), 105–112. https://doi.org/10.1016/j.brs.2013.08.006

Holtmann, J., Herbort, M. C., Wüstenberg, T., Soch, J., Richter, S., Walter, H., … Schott, B. H. (2013). Trait anxiety modulates fronto-limbic processing of emotional interference in borderline personality disorder. *Frontiers in Human Neuroscience*, *7*, 54. https://doi.org/10.3389/fnhum.2013.00054

Jacob, G. A., Zvonik, K., Kamphausen, S., Sebastian, A., Maier, S., Philipsen, A., … Tüscher, O. (2013). Emotional modulation of motor response inhibition in women with borderline personality disorder: an fMRI study. *Journal of Psychiatry & Neuroscience : JPN*, *38*(3), 164–172. https://doi.org/10.1503/jpn.120029

Korfine, L., & Hooley, J. M. (2000). Directed forgetting of emotional stimuli in borderline personality disorder. *Journal of Abnormal Psychology*, *109*(2), 214–221.

Krause-Utz, A., Oei, N. Y. L., Niedtfeld, I., Bohus, M., Spinhoven, P., Schmahl, C., & Elzinga, B. M. (2012). Influence of emotional distraction on working memory performance in borderline personality disorder. *Psychological Medicine*, *42*(10), 2181–2192. https://doi.org/10.1017/S0033291712000153

Krause-Utz, Annegret, Elzinga, B. M., Oei, N. Y. L., Spinhoven, P., Bohus, M., & Schmahl, C. (2014). Susceptibility to Distraction by Social Cues in Borderline Personality Disorder. *Psychopathology*, *47*(3), 148–157. https://doi.org/10.1159/000351740

Krause-Utz, Annegret, Winter, D., Niedtfeld, I., & Schmahl, C. (2014). The latest neuroimaging findings in borderline personality disorder. *Current Psychiatry Reports*, *16*(3), 438. https://doi.org/10.1007/s11920-014-0438-z

Lang, S., Kotchoubey, B., Frick, C., Spitzer, C., Grabe, H. J., & Barnow, S. (2012). Cognitive reappraisal in trauma-exposed women with borderline personality disorder. *NeuroImage*, *59*(2), 1727–1734. https://doi.org/10.1016/j.neuroimage.2011.08.061

Lieb, K., Zanarini, M. C., Schmahl, C., Linehan, M. M., & Bohus, M. (2004). Borderline personality disorder. *Lancet (London, England)*, *364*(9432), 453–461. https://doi.org/10.1016/S0140-6736(04)16770-6

Nigg, J. T., Wong, M. M., Martel, M. M., Jester, J. M., Puttler, L. I., Glass, J. M., … Zucker, R. A. (2006). Poor response inhibition as a predictor of problem drinking and illicit drug use in adolescents at risk for alcoholism and other substance use disorders. *Journal of the American Academy of Child and Adolescent Psychiatry*, *45*(4), 468–475. https://doi.org/10.1097/01.chi.0000199028.76452.a9

Nitsche, M. A., & Paulus, W. (2000). Excitability changes induced in the human motor cortex by weak transcranial direct current stimulation. *The Journal of Physiology*, *527 Pt 3*, 633–639.

Nitsche, M. A., & Paulus, W. (2001). Sustained excitability elevations induced by transcranial DC motor cortex stimulation in humans. *Neurology*, *57*(10), 1899–1901.

Nitsche, Michael A., Cohen, L. G., Wassermann, E. M., Priori, A., Lang, N., Antal, A., … Pascual-Leone, A. (2008). Transcranial direct current stimulation: State of the art 2008. *Brain Stimulation*, *1*(3), 206–223. https://doi.org/10.1016/j.brs.2008.06.004

Ochsner, K. N., & Gross, J. J. (2005). The cognitive control of emotion. *Trends in Cognitive Sciences*, *9*(5), 242–249. https://doi.org/10.1016/j.tics.2005.03.010

Prehn, K., Schulze, L., Rossmann, S., Berger, C., Vohs, K., Fleischer, M., … Herpertz, S. C. (2013). Effects of emotional stimuli on working memory processes in male criminal offenders with borderline and antisocial personality disorder. *The World Journal of Biological Psychiatry: The Official Journal of the World Federation of Societies of Biological Psychiatry*, *14*(1), 71–78. https://doi.org/10.3109/15622975.2011.584906

Schulze, L., Domes, G., Krüger, A., Berger, C., Fleischer, M., Prehn, K., … Herpertz, S. C. (2011). Neuronal correlates of cognitive reappraisal in borderline patients with affective instability. *Biological Psychiatry*, *69*(6), 564–573. https://doi.org/10.1016/j.biopsych.2010.10.025

Schulze, L., Schmahl, C., & Niedtfeld, I. (2016). Neural Correlates of Disturbed Emotion Processing in Borderline Personality Disorder: A Multimodal Meta-Analysis. *Biological Psychiatry*, *79*(2), 97–106. https://doi.org/10.1016/j.biopsych.2015.03.027

Sebastian, A., Jung, P., Krause-Utz, A., Lieb, K., Schmahl, C., & Tüscher, O. (2014). Frontal dysfunctions of impulse control - a systematic review in borderline personality disorder and attention-deficit/hyperactivity disorder. *Frontiers in Human Neuroscience*, *8*, 698. https://doi.org/10.3389/fnhum.2014.00698

Sieswerda, S., Arntz, A., Mertens, I., & Vertommen, S. (2007). Hypervigilance in patients with borderline personality disorder: specificity, automaticity, and predictors. *Behaviour Research and Therapy*, *45*(5), 1011–1024. https://doi.org/10.1016/j.brat.2006.07.012

Silbersweig, D., Clarkin, J. F., Goldstein, M., Kernberg, O. F., Tuescher, O., Levy, K. N., … Stern, E. (2007). Failure of frontolimbic inhibitory function in the context of negative emotion in borderline personality disorder. *The American Journal of Psychiatry*, *164*(12), 1832–1841. https://doi.org/10.1176/appi.ajp.2007.06010126

Soloff, P. H., Abraham, K., Ramaseshan, K., Burgess, A., & Diwadkar, V. A. (2017). Hyper-modulation of brain networks by the amygdala among women with Borderline Personality Disorder: Network signatures of affective interference during cognitive processing. *Journal of Psychiatric Research*, *88*, 56–63. https://doi.org/10.1016/j.jpsychires.2016.12.016

Wingenfeld, K., Rullkoetter, N., Mensebach, C., Beblo, T., Mertens, M., Kreisel, S., … Woermann, F. G. (2009). Neural correlates of the individual emotional Stroop in borderline personality disorder. *Psychoneuroendocrinology*, *34*(4), 571–586. https://doi.org/10.1016/j.psyneuen.2008.10.024

Wolkenstein, L., & Plewnia, C. (2013). Amelioration of cognitive control in depression by transcranial direct current stimulation. *Biological Psychiatry*, *73*(7), 646–651. https://doi.org/10.1016/j.biopsych.2012.10.010

Zetsche, U., Bürkner, P.-C., & Schulze, L. (2018). Shedding light on the association between repetitive negative thinking and deficits in cognitive control – A meta-analysis. *Clinical Psychology Review*, *63*, 56–65. https://doi.org/10.1016/j.cpr.2018.06.001

**Study goals and objectives**

This study aimed to advance our understanding of neural abnormalities in patients with borderline personality disorder. The primary objective of this study was to investigate whether anodal stimulation of the right dorsolateral prefrontal cortex ameliorates cognitive control of negative stimuli in patients with borderline personality disorder compared to a sham condition.

**Study Design and Methodology**

To address this research question, we enrolled 50 patients with borderline personality disorder (BPD) and 50 healthy controls between January 2016 and June 2017.

Healthy controls were only included if they did not take any psychotropic medication and had neither a current nor a lifetime diagnosis of any mental or neurological disorders (e.g., traumatic diseases of the central nervous system). Exclusion criteria for BPD patients were comorbid diagnosis of past or present psychotic disorder, current major depressive episode, bipolar disorder, cognitive disorders (e.g., delirium, dementia), or neurological disorders as well as substance-associated disorders within three months prior to study participation. In addition, we excluded participants with possible tDCS contraindications, such as a cardiac pacemaker, metal in or around the head, pregnancy, or tattoos or scarred skin on the scalp or left deltoid muscle.

Participants were assigned to receive either sham or verum stimulation of the right dorsolateral prefrontal cortex within a double-blind, between-subjects design. Direct electrical current was applied by a saline-soaked pair of surface sponge electrodes with a surface of 35mm² connected to a battery-driven constant current stimulator (DC-Stimulator, NeuroConn GmbH, Ilmenau, Germany). For anodal stimulation of the right dlPFC, the electrode was positioned over F4 according to the 10-20 international system for EEG electrode placement 42. The cathode was placed on the left deltoid muscle. During active stimulation a constant current of 1.0 mA was applied for the duration of the experimental paradigm (or a maximum of 20 minutes). To mimic the sensation of tDCS in the sham condition, the current was ramped up and down for 30 seconds respectively at the beginning and end of the experimental session. In the sham condition the stimulator was turned off during the experiment. The stimulation device contained a study mode for double-blind trials. The principal investigator generated numeric codes for active and sham stimulation sessions prior to the experimental sessions. Sequences were generated with in-house functions based on randperm (Matlab). The experimenter entered these preassigned codes and was unaware of the experimental condition.

During the stimulation period, participants performed a delayed working memory task. Each trial started with a fixation cross (1000 ms), followed by the presentation of six target letters (1500 ms), which participants were asked to memorize. After a variable distracter period (i.e., interference duration of 1000, 2000, or 4000 ms), participants were presented a recognition display (until a response was made) and had to decide whether the presented letter was part of the initial set of letters. In half of the trials, the recognition display contained a previously presented target. Participants were asked to respond as quickly and accurately as possible. The distracter period of the experimental paradigm was manipulated with regard to the factors valence (grey background screen, or neutral, or negative stimuli) and interference duration (1000, 2000, or 4000 ms). Neutral and negative affective stimuli were selected from the International Affective Picture System. In total, the experiment contained 180 trials, divided into nine blocks with 20 trials each. Each block contained a unique experimental condition (e.g., negative stimuli presented for 1000 ms). Visual stimuli in these blocks were matched regarding valence, arousal, luminance, and visual complexity (all p’s > .55). Experimental blocks were presented in pseudo-random order. The experiment was conducted on a standard notebook connected with a 15-inch screen (screen resolution of 1024 x 768).

**Safety considerations**

Participants were asked for the presence of possible side effects of the stimulation, i.e. perception of tingling or burning sensations, pain under the electrodes, light flashes during the stimulation, or headaches and nausea after the stimulation.

**Follow up**

This study had no follow-up assessment.

**Data management and statistical analysis**

We registered before the start of the experiment that we will calculated repeated-measures ANOVAs with reaction times or hit rates as dependent variable (cf. pre-registration at https://osf.io/g43bh/?view_only=f647fa67773041669f0a670c234dd150). These analyses comprise the within-subject factors valence (3 levels) and presentation times (3 levels) as well as the between-subject factors stimulation (2 levels) and diagnostic group (BPD and healthy controls). Our main hypothesis refered to a three-way interaction of valence, diagnostic group, and stimulation. In addition, we registered a secondary analysis with reaction times for negative stimuli that are accounted for reaction times in response to neutral stimuli. The pre-registration also specified the sample size of our study as well as performance-related exclusions of participants (hit rate below 65%).

#### Quality Assurance

The protocol should describe the quality control and quality assurance system for the conduct of the study, including GCP, follow up by clinical monitors, DSMB, data management etc.

**Expected outcomes of the study**

The results contribute to neurobiological models of affective disturbances in borderline personality disorder. In this study, we focused on the potential of brain stimulation in an experimental setting. Future studies might help to elucidate the potential of such applications for health care, health systems, or health policies.

**Dissemination of results**

Results will be disseminated to relevant psychotherapeutic and patient communities in peer-reviewed journals, and at scientific conferences

**Duration of the project**

The study took place at the Department of Psychiatry, Charité-Universitätsmedizin Berlin between January 2016 and June 2017.

**Problems anticipated**

We did not anticipate particular difficulties in carrying out this project.

**Project management**

L.S. and S.R. designed the study. S.T. programmed the experimental paradigm. M.G. contributed to implementation of the study and data collection. L.S. analyzed the data and wrote the first draft of the manuscript. M.G., B.R. and S.R. contributed to the interpretation of the data. All authors contributed to writing, reviewing and editing of the manuscript and approved the final version of the manuscript.

**Ethics**

The ethics committee of the Charité-Universitätsmedizin Berlin approved the study protocol and the study was conducted in accordance with the Helsinki Declaration. The research team members made sure that the study respected the following ethical principles: all the personal data gathered is treated confidentially, written informed consent was collected, data was securely stored, and the data will only be used for research purposes. Participation in this research study was voluntary. Participants were reminded of their rights to withdraw from the study without giving any reason. Data privacy was guaranteed: all the research data gathered during the project will be identified using pseudonyms. Personal data is kept under lock and stored separately from research data. Communications and publications will not enable identification of individual participants. All participants gave written informed consent prior to participation.

**Informed consent forms**

Informed consent forms will be made available upon request.

**Budget**

We did not apply for external funding of this project.

**Other support for the project**

Research position of Lars Schulze is funded by a grant from the German Research Foundation (DFG-SCHU 2961/2-1).
